# Metabolomic insights into *Monascus* ‐fermented rice products: Implications for monacolin K content and nutritional optimization

**DOI:** 10.1002/fsn3.4222

**Published:** 2024-05-13

**Authors:** Yongxia Zhao, Mingxia Luo, Qin Jiang, Yuhan Ma, Xiaoqi Liu, Xue Bai, Lihong Zhou, Jian Xie

**Affiliations:** ^1^ Department of Pharmacognostics Zunyi Medical University Zunyi China; ^2^ Guizhou Yuanxi Biological R & D Co., Ltd. Guiyang China; ^3^ Tibet Yuewang Medicine Diagnosis Ecological Tibetan Medicine Technology Co., Ltd. Lasha China; ^4^ School of Life Sciences Guizhou University Guiyang China; ^5^ Department of Medical Genetics Zunyi Medical University Zunyi China

**Keywords:** metabolomics, monacolin K, *Monascus*‐fermented rice products (MFRPs), sterilization methods

## Abstract

This study aims to elucidate the detailed metabolic implications of varying monacolin K levels and sterilization methods on *Monascus*‐fermented rice products (MFRPs), acclaimed for their health benefits and monacolin K content. Advanced metabolite profiling of various MFRP variants was conducted using ultrahigh‐performance liquid chromatography coupled with tandem time‐of‐flight mass spectrometry (UHPLC‐Q‐TOF MS). Statistical analysis encompassed *t*‐tests, ANOVA, and multivariate techniques including PCA, PLS‐DA, and OPLS‐DA. Notable variations in metabolites were observed across MFRPs with differing monacolin K levels, particularly in variants such as MR1‐S, MR1.5‐S, MR2‐S, and MR3‐S. Among the 524 identified metabolites, significant shifts were noted in organic acids, derivatives, lipids, nucleosides, and organic oxygen compounds. The study also uncovered distinct metabolic changes resulting from different sterilization methods and the use of highland barley as a fermentation substitute for rice. Pathway analysis shed light on affected metabolic pathways, including those involved in longevity regulation, cGMP‐PKG signaling, and the biosynthesis of unsaturated fatty acids. The research provides critical insights into the complex metabolic networks of MFRPs, underscoring the impact of fermentation substrates and conditions on monacolin K levels and their health implications. This study not only guides the nutritional optimization of MFRPs but also emphasizes the strategic importance of substrate choice and sterilization techniques in enhancing the nutritional and medicinal value of these functional foods.

## Graphical abstract

*Monascus*‐fermented rice products (MFRPs) are widely used in traditional and modern medicinal practices for their health benefits and monacolin K content, which is a natural statin that can lower cholesterol levels. However, the comprehensive metabolic impact of varying monacolin K levels and sterilization methods on MFRPs has not been fully elucidated.

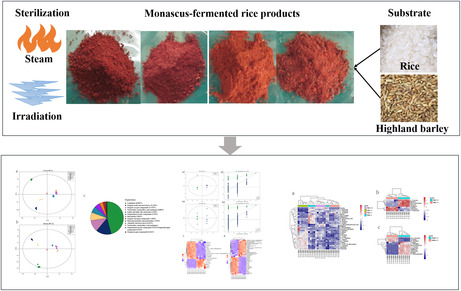

## 1 INTRODUCTION

*Monascus* fermentation products, especially those derived from solid‐state fermentation of rice, boast a rich heritage spanning over a millennium in China, serving dual roles as both nourishment and health supplements. In contemporary times, these *Monascus*‐fermented rice products (MFRPs) have garnered widespread acclaim as health foods, not only in the United States but also across various Asian nations, including Japan, China–Taiwan, Thailand, the Philippines, and Indonesia (Wang & Lin, 2007). Notably, these products were also applied in the dietary supplement Xuezhikang, a partially purified extract of fermented *Monascus* Rice. Launched in 2003, this supplement is a combination of 13 natural statins, unsaturated fatty acids, ergosterol, amino acids, flavonoids, alkaloids, trace elements, and other bioactive substances, garnering considerable research interest (Feng et al., 2012).

*Monascus* spp. can produce a diverse array of bioactive metabolites, such as polyketide monacolins, dimerumic acid, isoflavones, phytosterols, γ‐aminobutyric acid (GABA), and pigments (Ai et al., 2019). These metabolites are associated with numerous beneficial biological activities, including cholesterol modulation (Gerards et al., 2015), blood glucose regulation (Rajasekaran & Kalaivani, 2015), blood pressure management (Kuba et al., 2009), therapeutic benefits such as anticancer (Kurokawa et al., 2021), antioxidant (Agboyibor et al., 2018), and antilipid peroxidation (Yongxia et al., 2020), and prevention of Alzheimer's disease and osteoporosis (Lee & Pan, 2011).

Central to the efficacy of *Monascus*‐fermented rice products is lovastatin (monacolin K), which is identified as the principal bioactive compound with substantial pharmaceutical value (Lin et al., 2008; Patel, 2016). Monacolin K, the most potent among the naturally occurring monacolin forms of mevinolin (Xiong et al., 2019), exhibits varying concentrations of different MFRPs, such as MR1‐S, MR1.5‐S, MR2‐S, and MR3‐S, boasting monacolin K at average concentrations of 10.00, 15.00, 20.00, and 30.00 mg/g, according to different fermentation processes, respectively, each of which is amenable to high‐temperature steam sterilization. The efficacy of these MFRPs to regulate lipid balance appears to correlate with monacolin K content; however, the influence of concomitant metabolites amid changing monacolin K levels remains unclear. These concomitant metabolites might also exert significant physiological effects; hence, elucidating their alterations lays the groundwork for unraveling the broader pharmacological potential of MFRPs.

In addition, variants on the market, such as MR0.4‐F and MR1‐F, contain monacolin K concentrations of approximately 4.00 and 10.00 mg/g, respectively, but are subjected to radiation sterilization. In addition to high‐temperature sterilization, irradiation sterilization is also a commonly used method for food products. The advantages of the _60_Co‐γ ray irradiation technique are excellent penetrating power, no radioactive fallout, inexpensive processing costs, and can better preserve the nutrients in the material (Wang et al., 2023). High‐temperature steam sterilization is effective but can lead to the deterioration of heat‐sensitive substances, such as proteins and vitamins, potentially impacting the efficacy of the Chinese herbal medicines. On the other hand, _60_Co‐γ ray sterilization offers a simple and cost‐effective alternative for the conservation and sterilization of Chinese medicines (Huang et al., 2010). Numerous reports have shown that irradiation sterilization has less impact on the nutritional content of solid feed for animals or human food than high‐temperature sterilization and, more interestingly, may increase the content of polyphenols, flavonoids, and triterpenes in the substance (Shen et al., 2022). However, the impact of _60_Co‐γ radiation sterilization on the metabolites of *Monascus*‐Fermented Red Products (MFRPs) has not been extensively studied, and it is important to explore how this process differs from that of small molecule metabolites following steam sterilization.

The fermentation of MFRPs has seen new developments as customers seek nutrients and medicinal efficacy. Gradually, products of *Monascus* fermentation of other crops or herbal medicines have appeared on the market and in the laboratory (Huang et al., 2020; Zhao et al., 2021). Highland barley (*Hordeum vulgare*, Poaceae) (HB) is a nutritious crop in highland regions with excellent health benefits and shows promise as an economically important crop with diverse applications (Xie et al., 2023). HB offers superior nutritional value due to its lower starch content (Asare et al., 2011), higher protein content (Obadi et al., 2021), and wealth of vitamins, fibers, β‐glucans, bioactive carbohydrates, polyphenols, minerals, phenolics, and flavonoids (Šimić et al., 2019). The distinctive composition of highland barley contributes to a multitude of health benefits, including anti‐inflammatory, anticancer, antidiabetic, antibacterial, antiobesity, antifatigue, antiaging, hyperglycemic, and hyperlipidemic effects (Idehen et al., 2017; Song et al., 2020; Zhu et al., 2015). Highland barley *Monascus* represents a product resulting from the fermentation of highland barley seed by inoculation with *Monascus purpureus*, in contrast to the fermentation substrate *Monascus* Rice (Zhao et al., 2022). Therefore, when rice from the MFRP family is replaced with barley and *Monascus* is fermented, changes in small molecule metabolites must also be very interesting. Mass spectrometry‐based metabolomics approaches can enable the simultaneous detection and quantification of many metabolite features (Alseekh et al., 2021). Consequently, the main purpose of this article was to observe the changes in small metabolites in MFRPs with different monacolin K contents, changing the form of sterilization, and changing the fermentation substrate from a metabolomic perspective.

## 2 MATERIALS AND METHODS

### 2.1 Chemical reagents and MRP materials

All chemical reagents including ammonium acetate (NH_4_AC), ammonium hydroxide (NH_4_OH), ammonium fluoride (NH_4_F), and formic acid (FA) were sourced from Sigma‐Aldrich. Acetonitrile (CH_3_CN) was obtained from Merck. *Monascus*‐fermented rice products (MRPs) were procured from Zhejiang SANHE BIO‐TECH Co., Ltd.

The MFRPs are classified based on their targeted monacolin K content as part of our quality control and product specification: MR1‐S targets a monacolin K content of around 10.00 mg/g. MR1.5‐S targets around 15.00 mg/g. MR2‐S targets around 20.00 mg/g. MR3‐S targets around 30.00 mg/g. monacolin K content in each batch may vary slightly due to natural variations in the fermentation process. For example, MR1‐S typically ranges near 13.70 mg/g, MR1.5‐S around 15.85 mg/g, MR2‐S around 20.42 mg/g, and MR3‐S around 31.12 mg/g. Such variations are expected and are within an acceptable range that allows us to maintain consistency without significantly affecting the bioactivity of the product. These products are all subjected to high‐temperature steam sterilization, which can also influence the final monacolin K content, albeit minimally.

Additionally, MR0.4‐F and MR1‐F, with respective monacolin K concentrations of 4.83 mg/g and 11.02 mg/g, are processed using radiation sterilization. Similarly, MH0.4‐F, derived from highland barley and containing approximately 4.00 mg/g of Monacolin K, undergoes radiation irradiation sterilization.

The MR series represents a fermentation product obtained by fermenting rice with the fungus *Monascus*. Conversely, the MH series is derived from fermenting highland barley with the same *Monascus* species. The primary difference between these two series lies in their substrates—rice for MR and highland barley for MH—while all other fermentation conditions remain consistent. This distinction in substrates allows us to explore the influence of different grain types on the metabolomic profile produced by *Monascus* fermentation.

### 2.2 Sample collection and preparation

For sample preparation, 80 mg of *Monascus*‐fermented rice products (MFRPs) powder was initially thawed slowly at 4°C. To extract metabolites, 1000 μL of a methanol/acetonitrile/water mixture (2:2:1, v/v/v) was added. Following extraction, the mixture was centrifuged for 20 min at 14,000 × g at 4°C, and the supernatant was subsequently dried using a vacuum centrifuge. For further analysis, the dried samples were redissolved in 100 μL of an acetonitrile/water solution (1:1, v/v).

### 2.3 UHPLC‐Q‐TOF MS instrumentation and procedures

An ultrahigh‐performance liquid chromatography (UHPLC) system (1290 Infinity LC, Agilent Technologies) coupled to a quadrupole time‐of‐flight mass spectrometer (AB Sciex TripleTOF 6600) was utilized, facilitated by Shanghai Applied Protein Technology Co., Ltd. The analyses were conducted in both ESI‐positive and ESI‐negative ionization modes. The mobile phase consisted of component A (25 mM NH4AC and 25 mM NH4OH in water) and component B (CH3CN). The gradient began at 95% B for 0.5 min, linearly decreased to 65% over 7 min, then to 40% over the next minute, maintained for 1 min, then returned to 95% in 0.1 min, followed by a re‐equilibration period of 2.9 min. Quality control (QC) samples were interspersed within the sample set to evaluate system stability and data reliability.

For reversed‐phase liquid chromatography (RPLC) separation, the mobile phases in ESI‐positive mode were water with 0.1% formic acid (FA) as component A and CH3CN with 0.1% FA as component B. In ESI‐negative mode, component A was 0.5 mM NH4F in water. The gradient commenced at 1% B, increased linearly to 99% over 11.5 min, held for 3.5 min, and then returned to 1% B in 0.1 min with a re‐equilibration time of 3.4 min. The flow rate was set at 0.3 mL/min, and the column temperature was maintained at 25°C. Each sample was injected in 2‐μL aliquots.

Q‐TOF Mass Spectrometry Conditions: The ESI source conditions after HILIC chromatographic separation were as follows: Ion Source Gas1 (Gas1): 60; Ion Source Gas2 (Gas2): 60; Curtain gas (CUR): 30; Source temperature: 600°C; IonSapary Voltage Floating (ISVF) ± 5500 V; TOF MS scan m/z range: 60–1000 Da; Product IonScan m/z range: 25–1000 Da; TOF MS scan accumulation time: 0.20 s/spectra; and product ion scan accumulation time: 0.05 s/spectra.

### 2.4 Statistical analysis

Raw mass spectrometry data files (wiff.scan) were converted to MzXML format using ProteoWizard's MSConvert tool. The converted files were then imported into the XCMS software for further processing. Postnormalization to the total peak intensity, the data were analyzed using the R software package. This analysis included multivariate data techniques such as Pareto‐scaled principal component analysis (PCA) and orthogonal partial least‐squares discriminant analysis (OPLS‐DA). In the OPLS‐DA model, the variable importance in projection (VIP) scores was calculated for each metabolite to assess their contribution to the model's classification power. Additional chemometric analyses were conducted using MetaboAnalyst 5.0 to further explore the data. An analysis of variance (ANOVA) was performed to identify significant differences among the groups of independent samples. Metabolites were considered significantly altered based on a fold change (FC) greater than 1.5 and a *p*‐value of less than .05 (Zhao et al., 2023). Subsequently, the significantly altered metabolites were visualized using volcano plots and heatmaps of hierarchical clustering in both negative and positive ion modes. To interpret the biological relevance of the differential metabolites, statistical enrichment analysis was conducted against the Kyoto Encyclopedia of Genes and Genomes (KEGG) pathways (http://www.genome.jp/kegg/).

## 3 RESULTS

### 3.1 Metabolite identification

**FIGURE 1:**
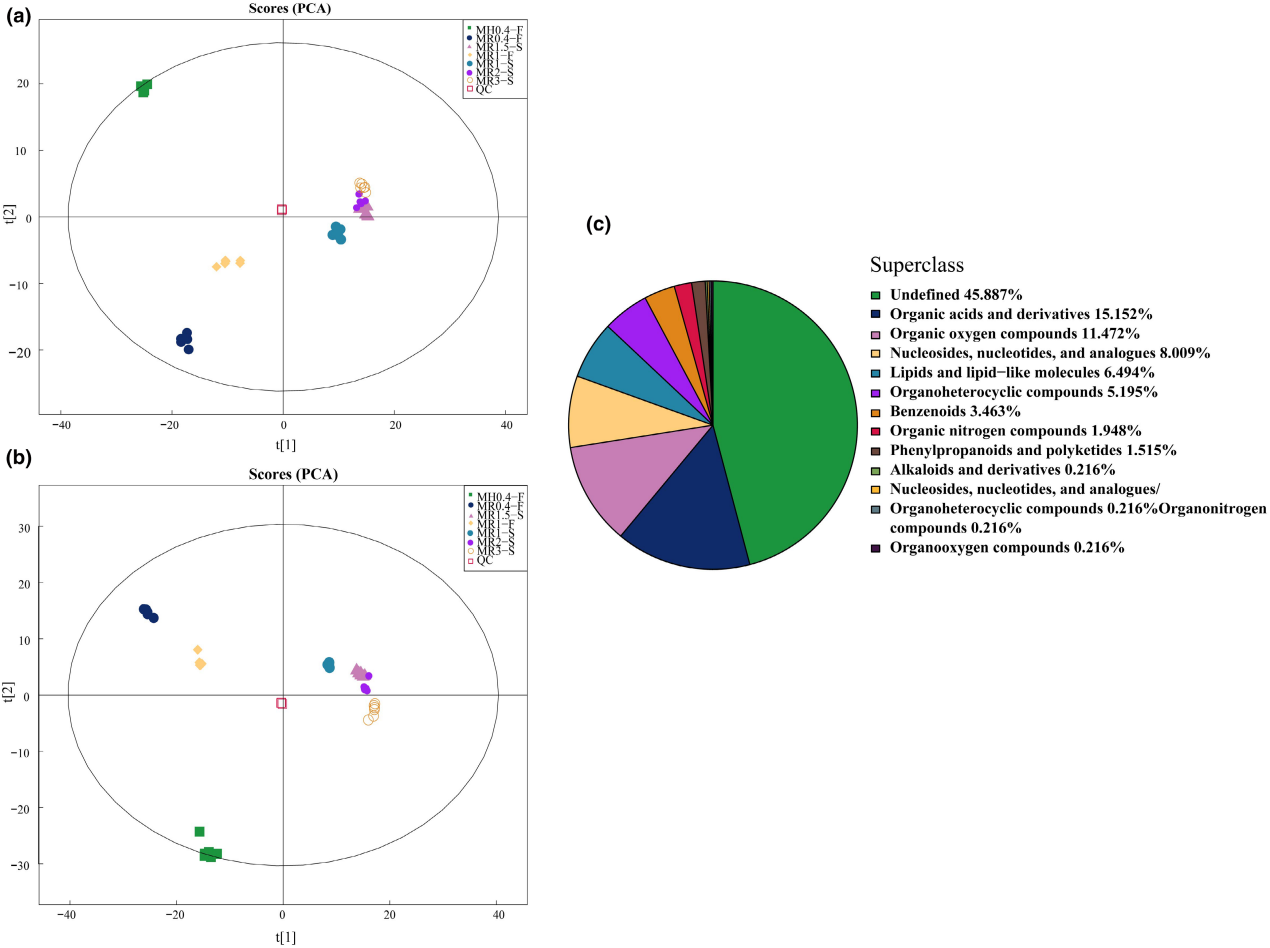
PCA and metabolite identification of all the samples. (a) PCA in positive ion mode (ESI+); (b) PCA in negative ion mode (ESI−); (c) percentage of identified metabolites in each chemical classification in terms of quantity.

In this study, we conducted metabolite profiling of various *Monascus*‐fermented rice products (MFRPs) using ultrahigh‐performance liquid chromatography coupled with tandem time‐of‐flight mass spectrometry (UHPLC‐Q‐TOF MS). All the identified metabolites were categorized into 13 superclasses, as illustrated in Figure 1c. Among these categories, the largest portion consisted of 212 unclassified metabolites, constituting 45.887% of the total identified metabolites. There were 70 organic acids and derivatives, accounting for 15.152% of the total. The third‐largest group comprised 53 organic oxygen compounds (11.472%), 37 nucleosides, nucleotides, and analogs (8.009%), 30 lipids and lipid‐like molecules (6.494%), 24 organoheterocyclic compounds (5.195%), and 16 benzenoids (3.463%). In summary, a total of 524 metabolites were identified across all the MFRP samples, including 297 metabolites from the ESI+ mode and 227 from the ESI‐ mode (Table S1).

The overall PCA score plots for samples in both positive ion mode (ESI+) and negative ion mode (ESI−) are depicted in Figure 1a,b, respectively. Notably, the QC samples clustered closely, indicating excellent experimental repeatability. Furthermore, samples MR1.5‐S, MR2‐S, MR3‐S, and MR1‐R were closely related, suggesting that the differences in metabolite composition among MR1‐S, MR1.5‐S, MR2‐S, MR3‐S, MR1‐F, MR0.4‐F, and MH0.4‐F were less pronounced. Our results also revealed significant variations among MR1.5‐S, MR2‐S, MR3‐S, MR1‐R, and the other samples, as they were dispersed in distinct regions within the PCA model.

### 3.2 Relevance of small molecule metabolites to monacolin K

**FIGURE 2:**
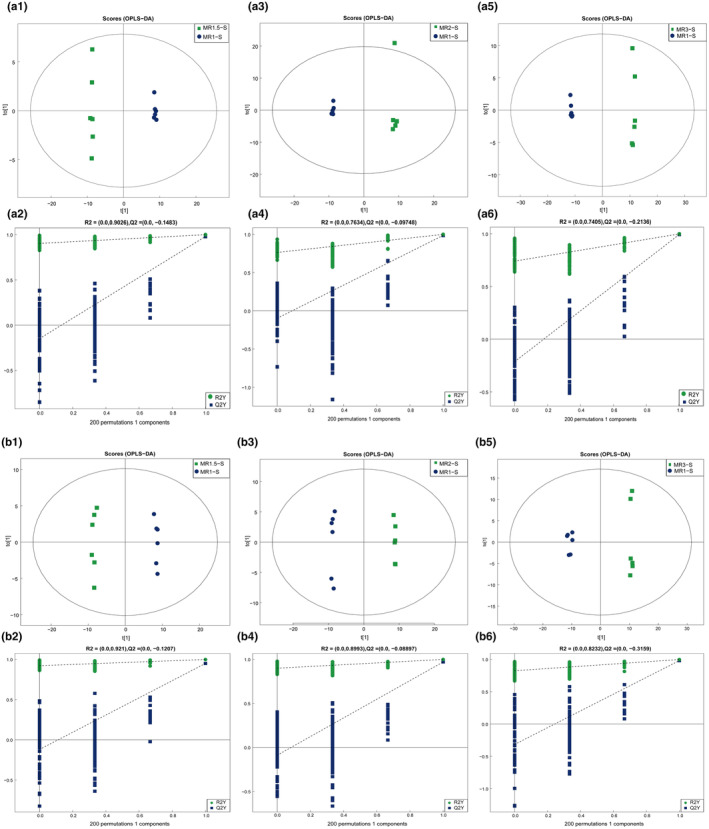
OPLS‐DA results for MFRPs with different monacolin K contents and replacement concentrations. (a1) In negative ion mode, OPLS‐DA between MR1.5‐S and MR1‐S; (a2) In negative ion mode, the replacement test of OPLS‐DA between MR1.5‐S and MR1‐S. The green dot represents R2, the blue dot represents Q2, and the two dotted lines represent the regression lines of R2 and Q2; (a3) In negative ion mode, OPLS‐DA between MR2‐S and MR1‐S; (a4) The replacement test between MR2‐S and MR1‐S; (a5) In negative ion mode, OPLS‐DA between MR3‐S and MR1‐S; (a6) The replacement test between MR3‐S and MR1‐S; (b1) In positive ion mode, OPLS‐DA between MR1.5‐S and MR1‐S; (b2) In positive ion mode, the replacement test of OPLS‐DA between MR1.5‐S and MR1‐S; (b3) In positive ion mode, OPLS‐DA between MR2‐S and MR1‐S; (b4) The replacement test between MR2‐S and MR1‐S; (b5) In positive ion mode, OPLS‐DA between MR3‐S and MR1‐S; (b6) The replacement test between MR3‐S and MR1‐S.

*Monascus*‐fermented rice products (MFRPs) undergo variations in monacolin K content during fermentation. To further investigate the differences in biochemical composition among MR1‐S, MR1.5‐S, MR2‐S, and MR3‐S, we performed orthogonal partial least squares discriminant analysis (OPLS‐DA). The OPLS‐DA score plot demonstrated that, compared with those in the MR1‐S group, the MR1.5‐S, MR2‐S, and MR3‐S groups exhibited significant metabolic changes within a 95% confidence interval (Figure 2). The results indicated the efficiency and reliability of the OPLS‐DA model, with intercepts of R2 and Q2 exceeding 0.95 in negative and positive ion modes, respectively (Table S2), as validated through cross‐validation using 12 components and 200 permutation simulations.

**FIGURE 3:**
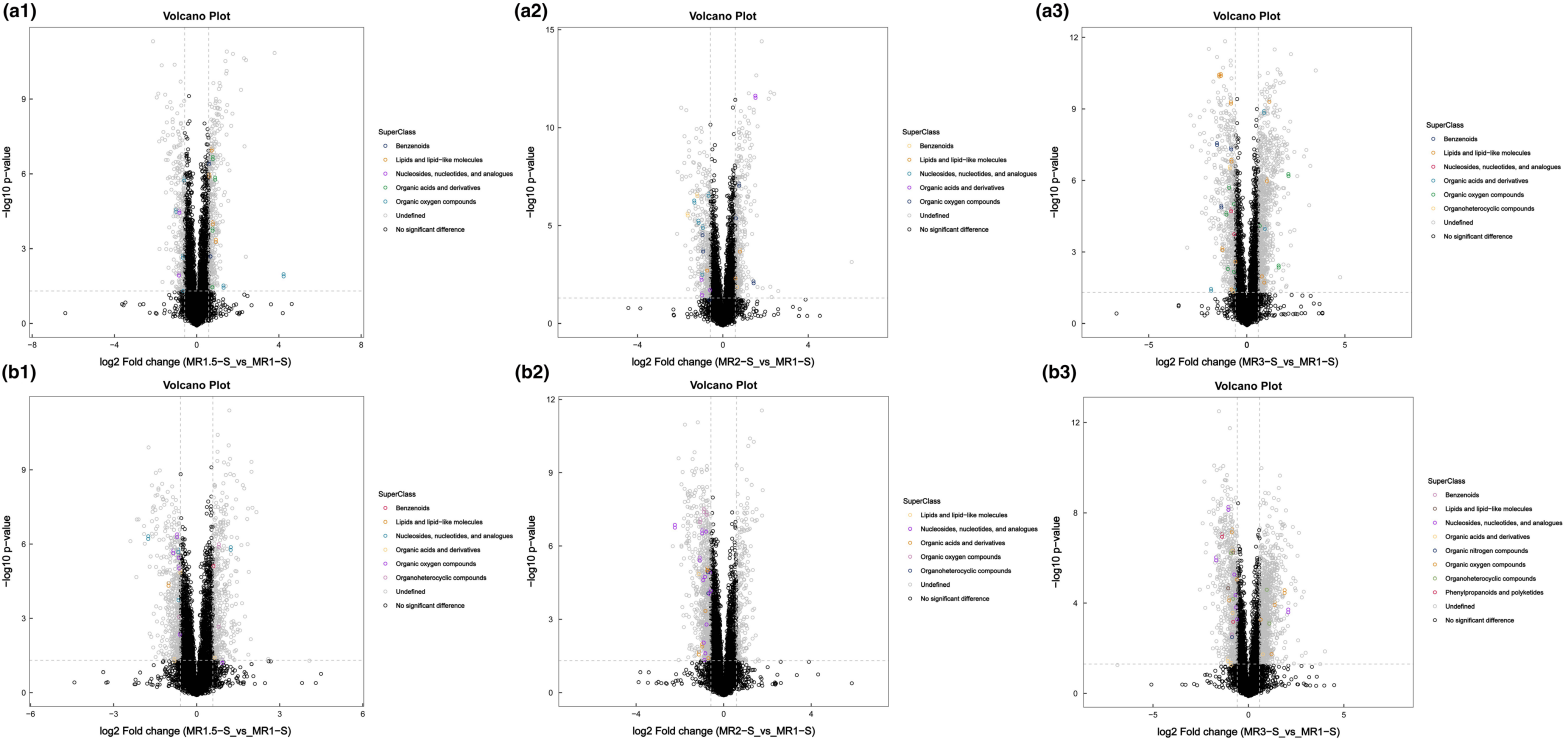
Volcano plots of MFRPs with different monacolin K contents under positive and negative ion conditions (FC > 1.5, *p* < .05). (a1) Volcano plots of MFRPs between MR1.5‐S and MR1‐S in negative ion mode; (a2) Volcano plots of MFRPs between MR2‐S and MR1‐S in negative ion mode. (a3): Volcano plots of MFRPs between MR3‐S and MR1‐S in negative ion mode; (b1) Volcano plots of MFRPs between MR1.5‐S and MR1‐S in positive ion mode. (b2): Volcano plots of MFRPs between MR2‐S and MR1‐S in positive ion mode; (b3) Volcano plots of MFRPs between MR3‐S and MR1‐S in positive ion mode.

To further analyze the differentially abundant metabolites between the samples and their correlation with monacolin K, we conducted a comparison between the MR1.5‐S, MR2‐S, and MR3‐S groups and MR1‐S, the results of which are visually presented as volcano diagrams (Figure 3). Notably, we identified small molecule metabolites that were significantly upregulated (*p* < .05) in MR1.5‐S compared to MR1‐S, including lipids and lipid‐like molecules, organic acids and derivatives, and organic oxygen compounds, in negative ion mode. Conversely, benzenoids, nucleosides, nucleotides, and analogs were downregulated (Figure 3a1). In the positive ion mode, the variation in the compounds was more extensive and irregular (Figure 3b1).

A similar trend was observed when comparing MR2‐S to MR1‐S. In the negative ion mode, lipids, lipid‐like molecules, organic oxygen compounds, and benzenoids were significantly upregulated, while organic acids and derivatives and nucleosides, nucleotides, and analogs were downregulated (Figure 3a2). In positive ion mode, a substantial number of compounds exhibited downregulation (Figure 3b2).

By comparing MR3‐S to MR1‐S in negative ion mode, we observed the downregulation of benzenoids, nucleosides, nucleotides, and analogs, while other superclass metabolites were both upregulated and downregulated (Figure 3a3). In the positive ion mode, both significantly upregulated and downregulated metabolites, including organic oxygen compounds, organoheterocyclic compounds, and a subset of nucleosides, nucleotides, and analogs, increased. Furthermore, phenylpropanoid and polyketide information was added to the list of downregulated metabolite species (Figure 3b3).

From the above analysis, it can be deduced that with increasing monacolin K content, other metabolites also undergo significant changes. Notably, the concentrations of organic oxygen compounds, lipids and lipid‐like molecules, and organic acids and their derivatives exhibited synergistic increases with the monacolin K content, providing valuable insights for subsequent analysis.

**FIGURE 4:**
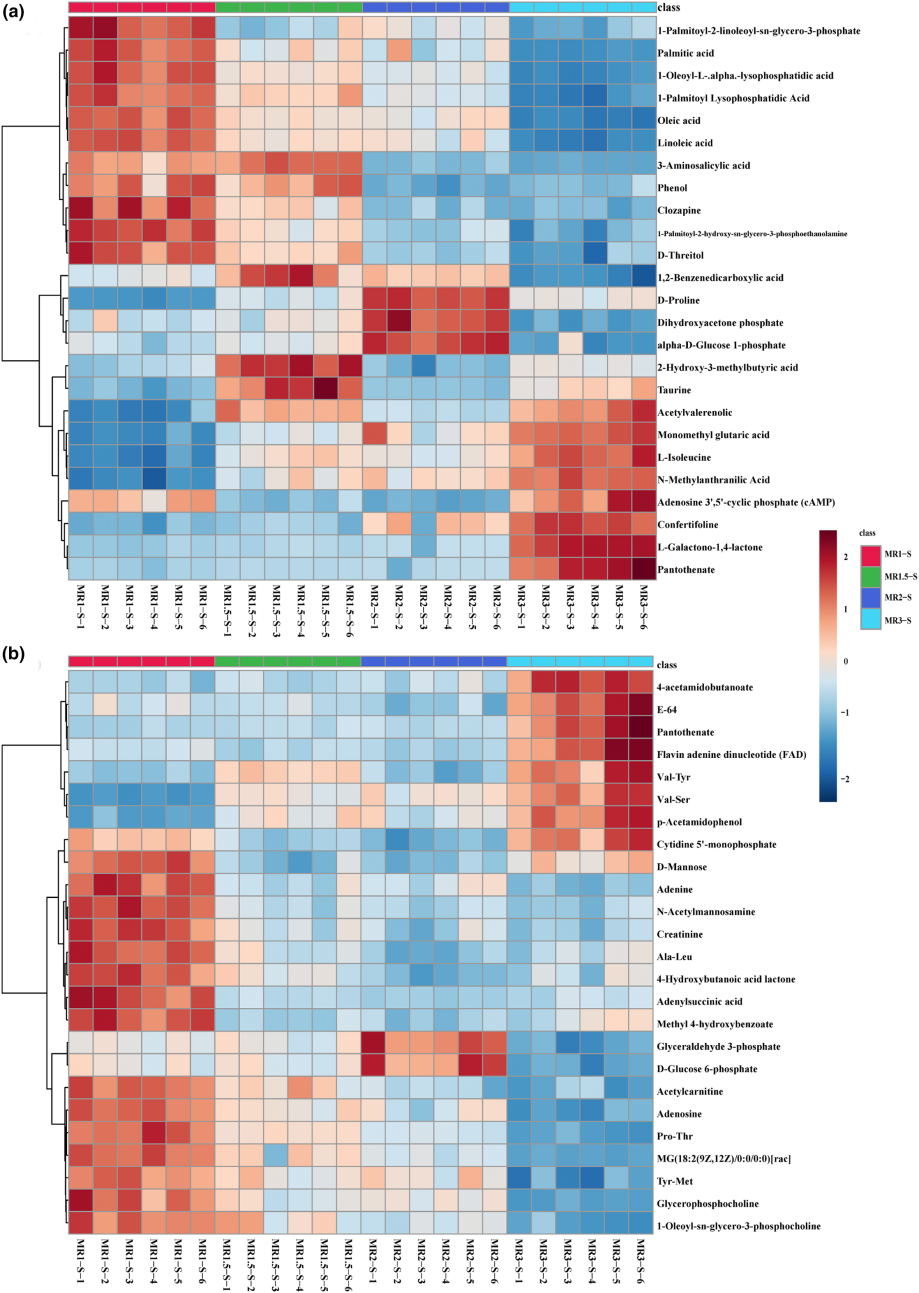
Heatmap of hierarchical clustering of significantly different metabolites among MR3‐S, MR2‐S, MR1.5‐S, and MR1‐S in negative and positive ion modes (a = neg, b = pos).

To identify differentially abundant metabolites among samples with varying monacolin K contents (MR1‐S, MR1.5‐S, MR2‐S, and MR3‐S), we conducted an analysis of variance (ANOVA) with *p* values <.05. We identified 139 differentially abundant metabolites in negative ion mode and 161 in positive ion mode (Table S3). Heatmap clustering analysis of the top 25 metabolites in negative ion mode revealed metabolites that exhibited trends similar to those of monacolin K, such as monomethyl glutaric acid (M167T51), L‐isoleucine (M130T220_2), N‐methylanthranilic acid (M210T250), adenosine 3′,5′‐cyclic phosphate (cAMP) (M328T269), and confusifoline (M467T61). Conversely, metabolites like 1‐palmitoyl‐2‐linoleoyl‐sn‐glycero‐3‐phosphate (M671T191_2), palmitic acid (M255T109), 1‐oleoyl‐L‐.alpha.‐lysophosphatidic acid (M435T245), 1‐palmitoyl lysophosphatidic acid (M409T249), oleic acid (M281T48), linoleic acid (M279T49) exhibited opposite trends (Figure 4a). In the positive ion mode, metabolites with similar trends to those of monacolin K were limited, including Val‐Ser (M205T245) and p‐acetylprophenol (acetaminophen, tylenol) (M152T372_2); however, other metabolites exhibited opposite trends, including acetylcarnitine (M204T348_1), adenosine (M268T174), Pro‐Thr (M234T348), MG (18:2(9Z,12Z)/0:0/0:0)[rac] (M337T241_2), Tyr‐Met (M427T439), glycerophosphocholine (M258T425_2), and 1‐oleoyl‐sn‐glycero‐3‐phosphocholine (M522T191_2) (Figure 4b).

The heatmap clustering analysis highlighted that metabolites with similar or opposite trends, both in positive and negative ion modes, were predominantly lipids and lipid‐like molecules; organic acids and derivatives; and nucleoside, nucleotide, and analog superclasses. Among the various types of differentially abundant metabolites observed in MFRPs with varying monacolin K levels, lipids and lipid‐like molecules emerged as particularly significant and biologically meaningful and are potentially linked to the synthetic pathway of monacolin K.

### 3.3 Effect of sterilization on small molecule metabolites of MFRPs

**FIGURE 5:**
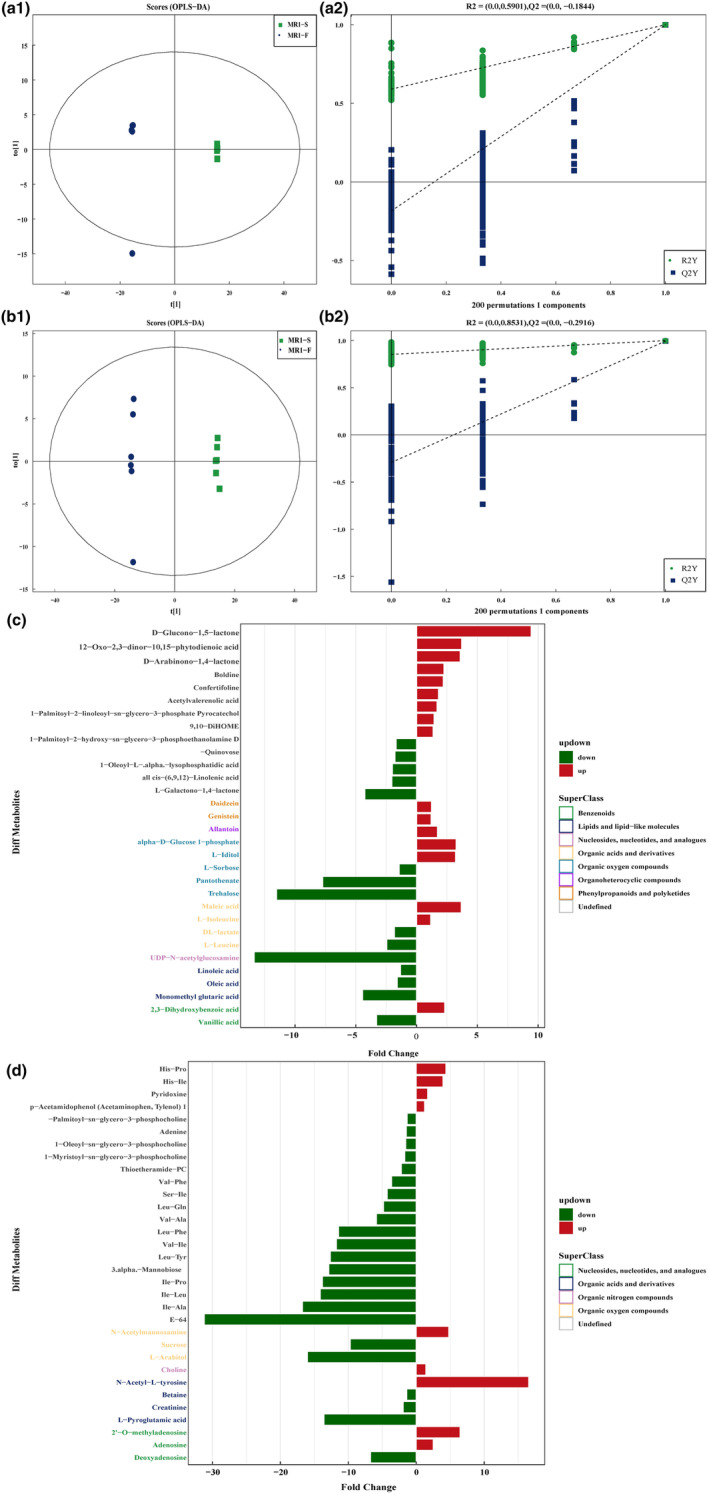
OPLS‐DA of MR1‐S versus MR1‐F with different sterilization methods and replacement tests and differential fold analysis of significant differentially abundant metabolite expression; (a1) In negative ion mode, OPLS‐DA between MR1‐S and MR1‐F; (a2) In negative ion mode, the replacement test of OPLS‐DA between MR1‐S and MR1‐F. The green dot represents R2, the blue dot represents Q2, and the two dotted lines represent the regression lines of R2 and Q2; (b1) In positive ion mode, OPLS‐DA between MR1‐S and MR1‐F; (b2) In positive ion mode, the replacement test of OPLS‐DA between MR1‐S and MR1‐F; (c) differential fold analysis of significant differentially abundant metabolite expression in negative ion mode; (d) differential fold analysis of significant differentially abundant metabolite expression in positive ion mode.

We examined the metabolic changes that occurred after altering the sterilization method of MFRPs with a monacolin K content of 10 mg/g. Significantly different metabolites emerged after both steam and radiation sterilization. In negative ion mode, the orthogonal partial least squares discriminant analysis (OPLS‐DA) model yielded the statistical parameters R2X (cum) = 0.861, R2Y (cum) = 1, and Q2 (cum) = 0.999 (Figure 5a1); however, in positive ion mode, these parameters were R2X (cum) = 0.72, R2Y (cum) = 0.999, and Q2 (cum) = 0.993 (Figure 5b1), confirming the reliability of the models (Table S2). Moreover, the replacement test (Figure 5a2,b2) ensured that the models were not overfitted (Figure 5a2,b2).

By comparing MR1‐S to MR1‐F, we identified 475 (neg) and 703 (pos) metabolites with VIP ≥1, for a total of 64 metabolites characterized among the differentially regulated metabolites (Table S4). In the negative ion mode, MR1‐S versus MR1‐F, 17 metabolites were significantly upregulated, including phenylpropanoid and polyketide compounds (daidzein and genistein), organoheterocyclic compounds (allantoin), organic oxygen compounds (alpha‐D‐glucose 1‐phosphate and L − iditol), organic acids and derivatives (maleic acid and L − isoleucine), and benzenoids (2,3 − dihydroxybenzoic acid). Additionally, 15 metabolites were significantly downregulated, comprising organic oxygen compounds (pantothenate, L‐sorbose, trehalose), organic acids and derivatives (DL‐lactate, L‐leucine), nucleosides, nucleotides, and analogs (UDP‐N‐acetylglucosamine); lipids and lipid‐like molecules (linoleic acid, oleic acid, monomethyl glutaric acid); and benzenoids (vanillic acid), along with five unclassified metabolites (Figure 5c).

In the positive ion mode, only nine metabolites were significantly upregulated, whereas the remainder were significantly downregulated. The upregulated compounds included N‐acetylmannosamine, choline, N‐acetyl‐L‐tyrosine, 2′‐O‐methyladenosine, and adenosine, whereas the 17 downregulated compounds mainly included amino acids and phosphocholines. Notably, the organic oxygen compounds sucrose and L‐Arabitol, as well as organic acids and derivative metabolites (betaine, creatinine, and L‐Arabitol), were significantly downregulated. Pyroglutamic acid and the nucleosides, nucleotides, and analog metabolite deoxyadenosine were also among the downregulated compounds (Figure 5d).

These results showed that the transition from steam sterilization to irradiation sterilization had a significant impact on the metabolites of MFRPs, primarily affecting lipids and lipid‐like molecules, as well as amino acids, which are vital nutrients in MFRPs.

### 3.4 Changes in *Monascus* highland barley products and *Monascus* rice products

**FIGURE 6:**
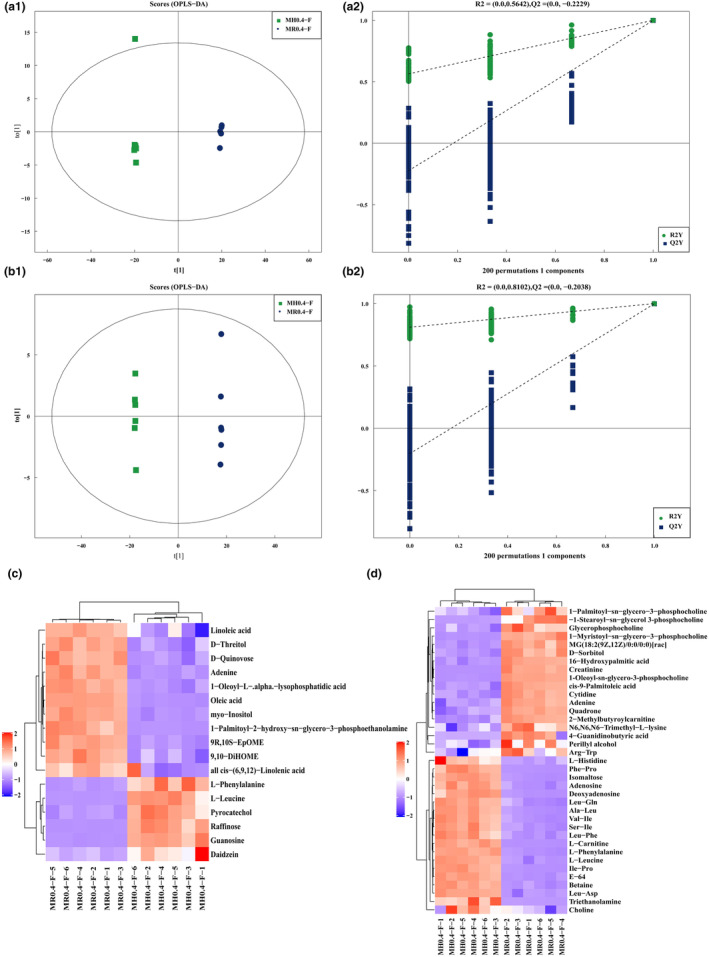
OPLS‐DA of MH0.4‐F and MR0.4‐F and replacement test and differential expression metabolites. (a1) In negative ion mode, OPLS‐DA between MH0.4‐F and MR0.4‐F; (a2) In negative ion mode, the replacement test of OPLS‐DA between MH0.4‐F and MR0.4‐F. The green dot represents *R* ^2^, the blue dot represents Q2, and the two dotted lines represent the regression lines of *R* ^2^ and *Q* ^2^; (b1) In positive ion mode, OPLS‐DA between MH0.4‐F and MR0.4‐F; (b2) In positive ion mode, the replacement test of OPLS‐DA between MH0.4‐F and MR0.4‐F; (c) The differential expression metabolites of MH0.4‐F versus MR0.4‐F in negative ion mode; (d) The differential expression metabolites of MH0.4‐F versus MR0.4‐F in positive ion mode.

Our analysis revealed significant differences in metabolites following substitution, with the statistical parameters of the orthogonal partial least squares discriminant analysis (OPLS‐DA) models being R2X (cum) = 0.91, R2Y (cum) = 1, and Q2 (cum) = 0.999 in negative ion mode (Figure 6a1) and R2X (cum) = 0.808, R2Y (cum) = 1, and Q2 (cum) = 0.998 in positive ion mode (Figure 6b1) (Table S2), underscoring the reliability of the models. The replacement test for model quality (Figure 6a2,b2) further confirmed that the models were not overfitted.

Following OPLS‐DA, metabolites with a VIP ≥1 and *p* < .05 were selected for further examination. In the negative ion mode, 17 metabolites exhibited significant differences, with six metabolites significantly elevated in MH0.4‐F compared to MR0.4‐F. These included L‐phenylalanine, L‐leucine, pyrocatechol, raffinose, guanosine, and daidzein. In contrast, 11 compounds, namely, linoleic acid, D‐threitol, D‐quinovose, adenine, 1‐oleoyl‐L‐alpha‐lysophosphatidic acid, oleic acid, myo‐inositol, 1‐palmitoyl‐2‐hydroxy‐sn‐glycero‐3‐phosphatidic acid, 9R, 10S‐EpOME, and 9,10‐DiHOME, all of which were cis‐(6,9,12)‐linolenic acid (Figure 6c), were significantly decreased.

In the positive ion mode, the number of differentially abundant metabolites was greater. Among the 37 significantly differentially abundant metabolites, those upregulated by MH0.4‐F included L‐histidine, Phe‐Phe, isomaltose, adenosine, deoxyadenosine, Leu‐Gln, Ala‐Leu, Val‐Ile, Ser‐Ile, Leu‐Phe, L‐carnitine, L‐phenylalanine, L‐leucine, Ile‐Pro, E‐64, betaine, Lue‐Asp, triethanolamine, and choline. These metabolites encompassed organic acids and derivatives, organic nitrogen compounds, organic oxygen compounds, and nucleoside, nucleotide, and analog superclasses. Conversely, 18 metabolites were significantly downregulated by MH0.4‐F, including 1‐palmitoyl‐sn‐glycero‐3‐phosphocholine, 1‐stearoyl‐sn‐glycerol, 3‐phosphocholine, glycerophosphocholine, 1‐myristoyl‐sn‐glycero‐3‐phosphocholine, MG (18:2(9Z,12Z)/0:0/0:0), D‐sorbitol, 16‐hydroxypalmitic acid, creatinine, 1‐oleoyl‐sn‐glycero‐3‐phosphocholine, cis‐9‐palmitoleic acid, cytidine, adenine, quadrone, 2‐methylbutyroylcarnitine, N6,N6,N6‐trimethyl‐L‐lysine, 4‐guanidinobutyric acid, perillyl alcohol, and Arg‐Trp. These metabolites belonged to the organic acids and derivatives, lipids and lipid‐like molecules, nucleosides, nucleotides, and analogs, and organic oxygen compound superclasses (Figure 6d).

### 3.5 Pathway analysis of differentially abundant metabolites

**FIGURE 7:**
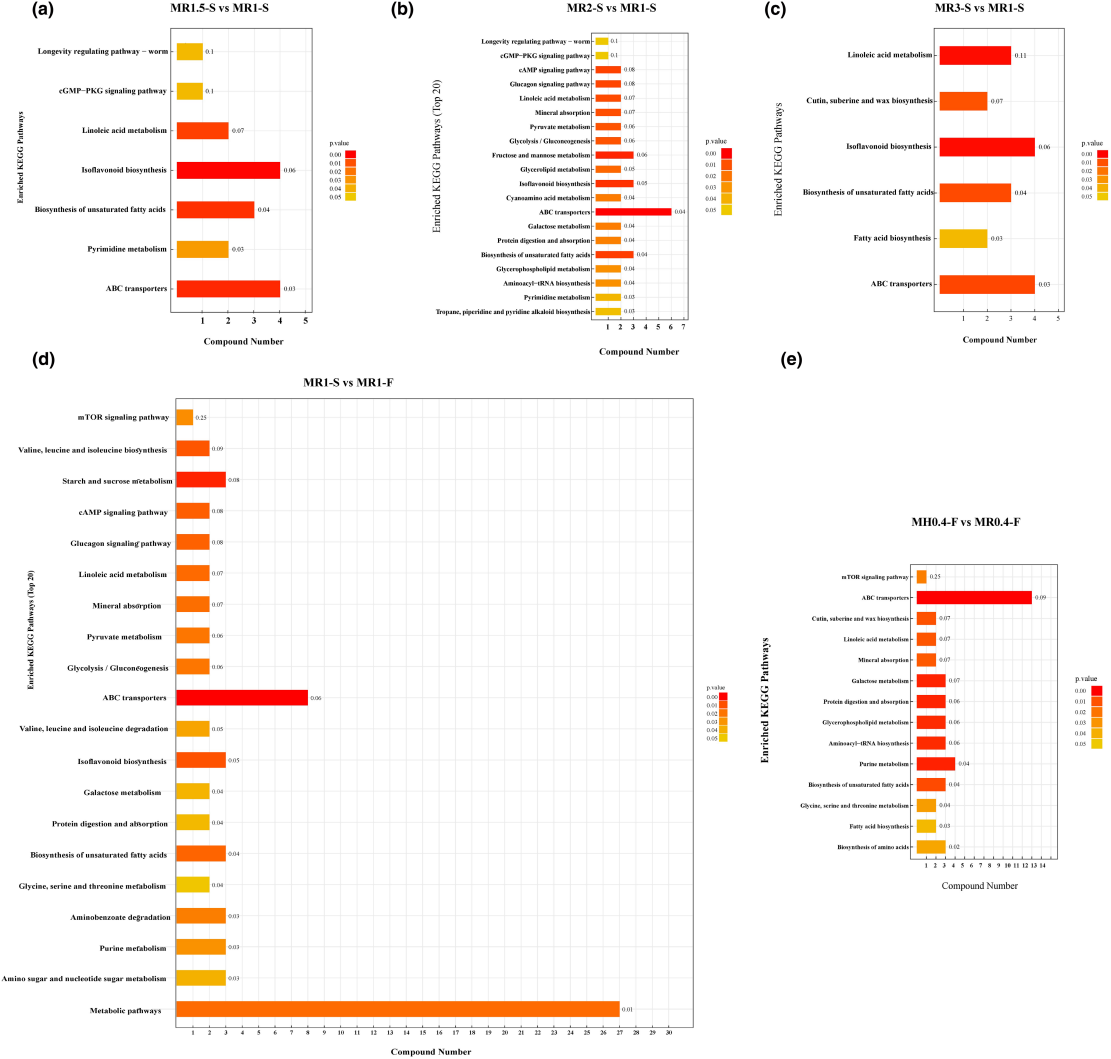
KEGG enrichment pathway of all samples. (a) KEGG enrichment pathway of MR1.5‐S and MR1‐S; (b) KEGG enrichment pathway of MR2‐S and MR1‐S; (c) KEGG enrichment pathway of MR3‐S and MR1‐S; (d) KEGG enrichment pathway of MR1‐S and MR1‐F; (e) KEGG enrichment pathway of MH0.4‐F and MR0.4‐F.

To investigate the influence of differentially abundant metabolites on metabolic pathways, Fisher's exact test was used to analyze and calculate the significance level of metabolite enrichment for each pathway, thus identifying the metabolic and signal transduction pathways that were significantly affected. The differentially abundant metabolites between MR1.5‐S and MR1‐S were enriched mainly in the longevity regulating pathway, cGMP‐PKG signaling pathway, linoleic acid metabolism, isoflavonoid biosynthesis, biosynthesis of unsaturated fatty acids, pyrimidine metabolism, and ABC transporters (Figure 7a). There were more differentially abundant metabolites between MR2‐S and MR1‐S than between the other MR2‐S groups, and the top 20 KEGG pathways enriched in ABC transporters, fructose and mannose metabolism, biosynthesis of unsaturated fatty acids isoflavonoid biosynthesis, linoleic acid metabolism, longevity regulating pathway, cGMP‐PKG signaling pathway, and so on were enriched. Furthermore, differentially abundant metabolites between MR3‐S and MR1‐S were enriched in six main KEGG pathways: linoleic acid metabolism; cutin, suberin and wax biosynthesis; isoflavonoid biosynthesis; biosynthesis of unsaturated fatty acids; fatty acid biosynthesis; and ABC transporter pathways (Figure 7c). A comparison of these three pairs revealed that the differentially abundant metabolites of the MFRPs with different monacolin K contents were mainly related to linoleic acid metabolism, the biosynthesis of unsaturated fatty acids and ABC transporters.

When the sterilization pattern after *Monascus* fermentation was altered, the differentially abundant metabolites were found to be mainly concentrated in metabolic pathways, where ABC transporters, biosynthesis of unsaturated fatty acids, and isoflavonoid biosynthesis were involved. Starch and sucrose metabolism were the major enrichment pathways (Figure 7d). Perhaps the irradiation sterilization method helps MFRPs retain more nutrients. When comparing MH0.4‐F with MS0.4‐F, it was found that the differentially abundant metabolites were mainly involved in the mTOR signaling pathway; ABC transporters; Cutin, suberin and wax biosynthesis; linoleic acid metabolism; mineral absorption; galactose metabolism; protein digestion and absorption; glycerophospholipid metabolism; aminoacyl−tRNA biosynthesis; purine metabolism; biosynthesis of unsaturated fatty acids; glycine, serine and threonine metabolism; fatty acid biosynthesis; and biosynthesis of amino acids (Figure 7e). These metabolic pathways include amino acid metabolism and synthesis, fatty acid metabolism and synthesis, and sugar metabolism; thus, the replacement of rice with barley affects the primary and secondary metabolism of the fermentation metabolites of MFRPs.

**FIGURE 8:**
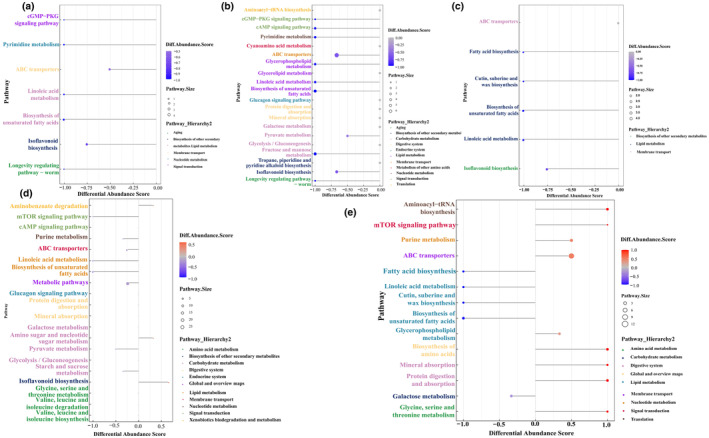
Differential abundance score plots for all the differential metabolic pathways. (a) Differential abundance score plots of MR1.5‐S and MR1‐S; (b) differential abundance score plots of MR2‐S and MR1‐S; (c) differential abundance score plots of MR3‐S and MR1‐S; (d) differential abundance score plots of MR1‐S and MR1‐F; (d) differential abundance score plots of MH0.4‐F and MR0.4‐F.

The average overall change in the abundance of all the metabolites among these pathways was demonstrated by the following differential abundance score. MR1.5‐S exhibited a downward trend in the average change in the abundance of all the compounds in the differentially abundant metabolite‐enriched metabolic pathways compared to MR1‐S; these pathways included ABC transporters, fatty acid biosynthesis, biosynthesis of unsaturated fatty acids, and linoleic acid metabolism pathways in lipid metabolism (Figure 8a). MR2‐S and MR3‐S exhibited the same trend as MR1‐S, which showed that with the upregulation of monacolin K, many metabolites were downregulated (Figure 8bc). In terms of metabolic pathways, the mTOR signaling pathway, linoleic acid metabolism, and biosynthesis of unsaturated fatty acids were significantly upregulated in MR1‐F after the change to radiation sterilization (Figure 8d). Thus, radiation has a retention effect on many metabolites. When *Monascus* rice was replaced with *Monascus* barley, the following metabolic pathways were downregulated: fatty acid biosynthesis, glycerophospholipid metabolism, biosynthesis of unsaturated fatty acids, cutin, suberin and wax biosynthesis, and linoleic acid metabolism pathways. Similarly, aminoacyl‐tRNA biosynthesis, the mTOR signaling pathway, purine metabolism, ABC transporters, biosynthesis of amino acids, mineral absorption, and protein digestion and absorption were significantly upregulated (Figure 8e).

### 3.6 ABC transporter pathway

**FIGURE 9:**
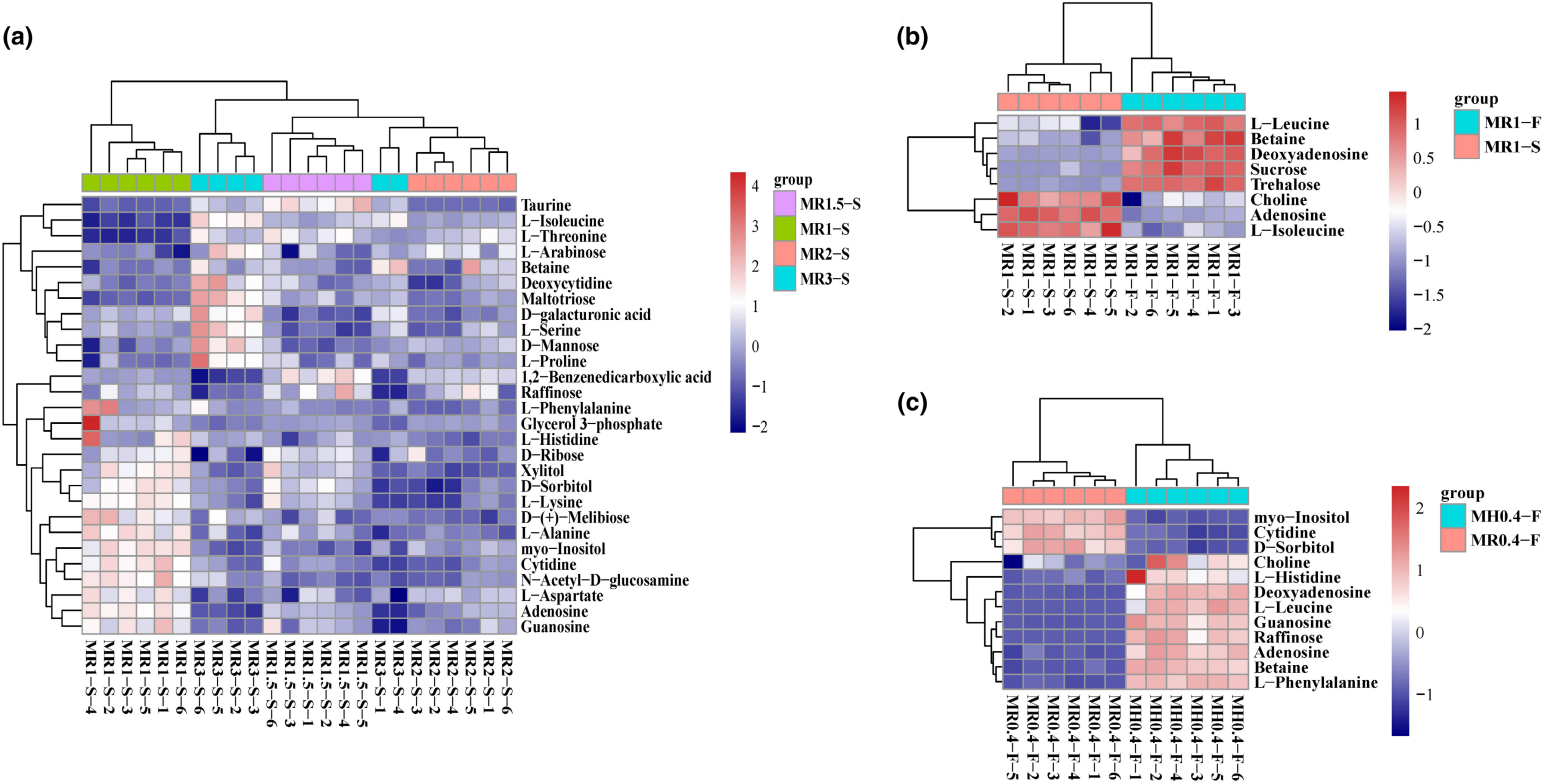
Clustering of differentially abundant metabolites in the ABC transporter pathway. (a) Differentially abundant metabolites of MR3‐S, MR2‐S, MR1.5‐S, and MR1‐S with different monacolin K contents in the ABC transporter pathway; (b) Differentially abundant metabolites of MR1‐S and MR1‐S with different sterilization methods in the ABC transporter pathway; (c) Differentially abundant metabolites of MR0.4‐F and MH0.4‐F in the rice or barley ABC transporter pathway.

Among the metabolites of the different MFRPs, the most significant metabolite changes were in the ABC transporter and biosynthesis of secondary metabolites pathways, which included many fatty acid metabolic pathways, such as biosynthesis of unsaturated fatty acids. By analyzing the metabolites that differed in terms of pathway, we found that among the ABC transporters, MR3‐S, MR2‐S, MR1.5‐S and MR1‐S had small amounts of metabolites, such as xylitol, D‐sorbitol, L‐lysine, D‐Melibiose, L‐alanine, myo‐inositol, cytidine, N‐acetyl‐D‐glucosamine, L‐aspartate, adenosine, and guanosine, whereas many of the small differentially abundant metabolites were not significantly elevated in the MFRPs with high monacolin K content (Figure 9a). A number of transporters associated with monosaccharides, oligosaccharides, lipids, phosphoric acid, and amino acids are reduced as monacolin K is elevated (Table 1), affecting the mechanism of cell membrane transport.

**TABLE 1:** The differential metabolites in ABC transporter pathway.

Transporters	Metabolites	MR3‐S, MR2‐S, MR1.5‐S vs. MR1‐S	Metabolites	MR1‐F vs. MR1‐S	Metabolites	MH0.4‐F vs. MR0.4‐F
Monosaccharide transporters	Xylitol	Down				
	Myo‐inositol	Down				
Oligosaccharide, polyol, and lipid transporters	D − Sorbitol	Down	Deoxyadenosine	Up	Guanosine	Up
	D−(+) − Melibiose	Down	Sucrose	Up	Raffinose	Up
	Cytidine	Down	Trehalose	Up	Adenosine	Up
	N‐acetyl‐D‐glucosamine	Down	Adenosine	Down	Deoxyadenosine	Up
	Adenosine	Down				
	Guanosine	Down				
Phosphate and amino acid transporters	L‐Lysine	Down	L − Leucine	Up	L − Histidine	Up
	L‐Alanine	Down	L − Isoleucine	Down	L − Leucine	Up
	L‐Aspartate	Down				
Mineral and organic ion transporters			Betaine	Up	Betaine	Up
Mineral and organic ion transporters			Choline	Down	Choline	Up

L‐Leucine, betaine, deoxyadenosine, sucrose, and trehalose, which are ABC transporters, were significantly increased, and choline, adenosine, and L‐isoleucine were significantly decreased when the MFRP treatment was changed from steam sterilization to radiation sterilization (Figure 9b). When the fermentation substrate was changed from rice to barley, the contents of choline, L‐histidine, deoxyadenosine, L‐leucine, guanosine, raffinose, adenosine, betaine, and L‐phenylalanine increased significantly (Figure 9c).

### 3.7 Pathway of biosynthesis of secondary metabolites

**FIGURE 10:**
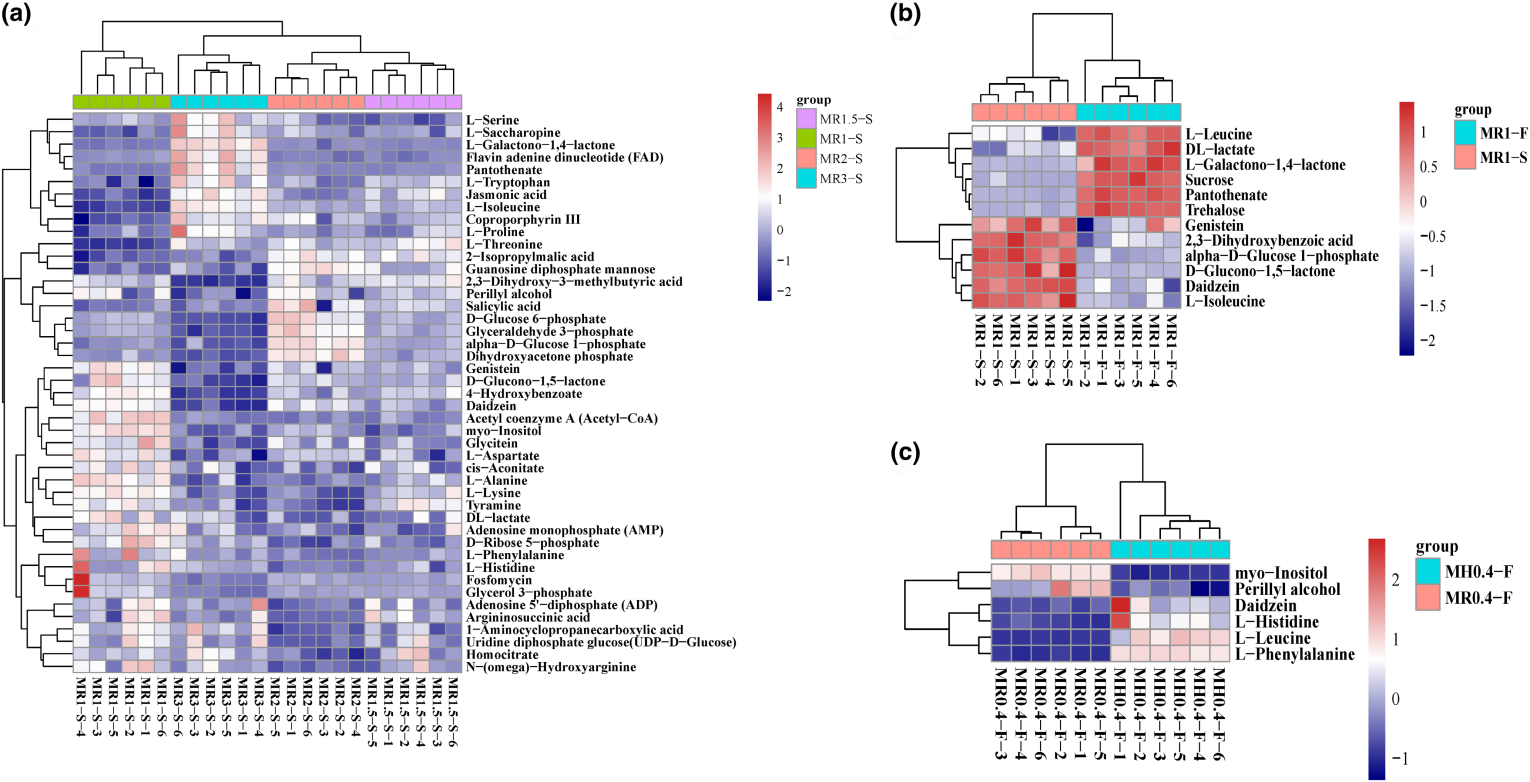
Clustering of differentially abundant metabolites involved in the biosynthesis of secondary metabolites pathway. (a) Differentially abundant metabolites of MR3‐S, MR2‐S, MR1.5‐S, and MR1‐S with different monacolin K contents in the biosynthesis of secondary metabolites pathway; (b) Differentially abundant metabolites of MR1‐S and MR1‐S with different sterilization methods in the biosynthesis of secondary metabolites pathway; (c) Differentially abundant metabolites of MR0.4‐F and MH0.4‐F with rice or barley in the biosynthesis of secondary metabolite pathway.

In the biosynthesis of secondary metabolites, a small number of metabolites, such as genistein, D‐glucono‐1,5‐lactone, 4‐hydroxybenzoate, daidzein, acetyl coenzyme A (acetyl‐CoA), myo‐inositol, glycitein, L‐aspartate, cis‐acrylate, L‐alanine, L‐lysine, and tyramine, were slightly more common in MR1‐S than in MR3‐S, MR2‐S, and MR1.5‐S. Moreover, the levels of L‐serine, L‐saccharopine, L‐galactono‐1,4‐lactone, flavin adenine, dinucleotide (FAD), pantothenate, and L‐tryptophan were greater in MR3‐S than in the other strains (Figure 10a). Hence, in the biosynthesis of secondary metabolites pathway, secondary metabolites exhibit both synergistic elevation and reduced expression, and the metabolic pathway is a holistic network and is, therefore, intricately regulated.

When the sterilization method of MR1 was changed from steam sterilization to radiation sterilization, the levels of L‐leucine, DL‐lactate, L‐Galactono‐1,4‐lactone, sucrose, pantothenate, and trehalose were significantly elevated, while the levels of genistein, 2,3‐dihydroxybenzoic acid, alpha‐D‐glucose 1‐phosphate, D‐Glucono‐1,5‐lactone, and Daidzein, L‐isoleucine were significantly lower (Figure 10b). Compared with those of *Monascus* rice products, the contents of daidzein, L‐histidine, L‐leucine, and L‐phenylalanine significantly improved, yet the contents of myo‐inositol and perillyl alcohol significantly decreased (Figure 10c).

## 4 DISCUSSION

A comprehensive analysis of the MFRPs revealed the presence of 524 metabolites, encompassing a diverse range of metabolites, including lipids and lipid‐like molecules; organic acids and their derivatives; organo‐oxygen compounds; nucleosides; nucleotides and analogs; organoheterocyclic compounds; phenylpropanoids and polyketides; benzenoids; alkaloids and derivatives; and organic nitrogen compounds. Key differentially abundant metabolites predominantly included proline and its derivatives, long‐chain fatty acids, aminosalicylic acids, linoleic acids and derivatives, isoleucine and derivatives, organosulfonic acids, hydroxy fatty acids, monosaccharide phosphates, 3′,5′‐cyclic purine nucleotides, 1‐hydroxy‐4‐unsubstituted benzenoids, fatty acid methyl esters, pyrimidine nucleotide sugars, sugar alcohols, isoflavones, hydroxybenzoic acid derivatives, medium‐chain fatty acids, purine ribonucleoside monophosphates, glycerophosphocholines, secondary alcohols, glyceraldehyde‐3‐phosphates, purine nucleosides, acylaminosugars, hexose phosphates, hexoses, pyrimidine 2′‐deoxyribonucleoside diphosphates, acyl carnitines, pyridine carboxylic acids, isoflavonoid O‐glycosides, and pyrimidine ribonucleoside diphosphates. Notably, an increase in monacolin K levels corresponded with a synergistic increase in organic oxygen compounds, lipids, lipid‐like molecules, and organic acids and derivatives.

**FIGURE 11:**
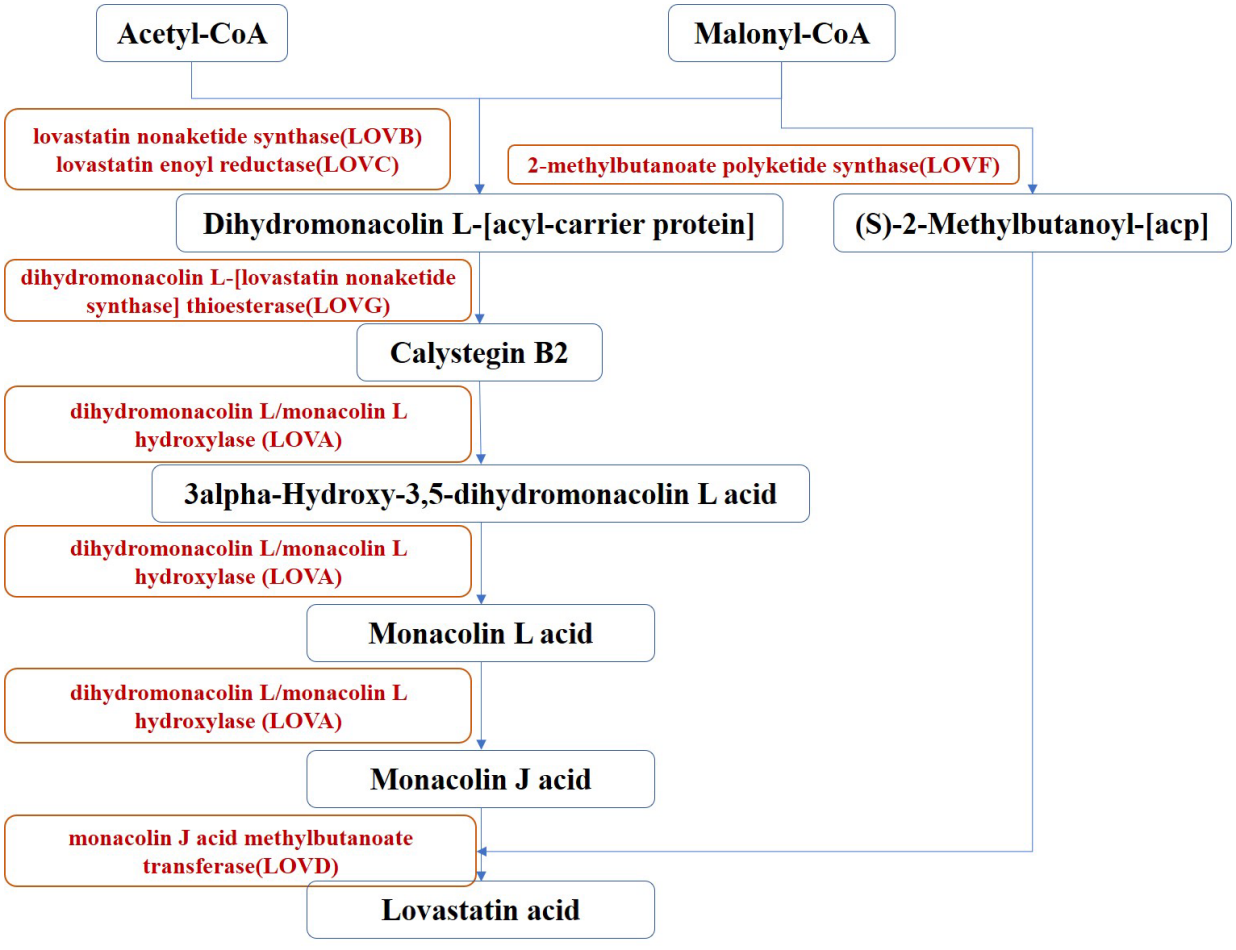
The synthetic pathway of monacolin K.

Monacolin K, known for its efficacy in treating hypercholesterolemia and referred to as lovastatin or Mevastatin (Da Porto et al., 2023), is synthesized from acetyl coenzyme A and malonyl coenzyme A through a complex process involving multiple enzymes (Campbell & Vederas, 2010). This synthesis pathway starts with the formation of dihydromonacolin L., which is facilitated by the polyketide synthase encoded by LovB in the presence of the enoyl reductase LovC (Figure 11). This initial step involved eight polyketide synthetic cycles encompassing approximately 35 steps (Wang et al., 2021). Subsequent stages involve the action of the thioesterase LovG (Xu et al., 2013), the cytochrome P450 enzyme LovA, and other enzymes, such as LovD and LovF, each of which contributes to the gradual transformation of dihydromonacolin L into lovastatin (Roth, 2002). A metabolic shift toward monacolin K synthesis significantly affects the production of other small molecule metabolites, notably influencing the fatty acid metabolic pathway and providing insights for enhancing monacolin K production.

Although effective, conventional high‐temperature and high‐pressure sterilization methods can detrimentally alter the nutritional and medicinal properties of food and pharmaceutical products (Zhang et al., 2024). In contrast, irradiation sterilization, utilizing γ‐rays from a Co_60_ radiation source, offers a gentler approach, preserving volatile components and heat‐sensitive substances in MFRPs. Studies have shown that irradiation is more effective at retaining medicinal properties than moist heat sterilization (Laurent et al., 2023). The differential regulation of 64 metabolites in MFRPs poststerilization underscores the need for careful consideration of sterilization methods to maintain the integrity and nutritional value of these products.

Traditionally, MFRPs are produced using rice as the substrate. However, recent advancements have introduced a variety of substrates, including medicinal herbs and other grains, to cater to a broader range of pharmaceutical and nutraceutical needs (Huang et al., 2020; C. Zhao et al., 2021). Fermentation with substrates such as hawthorn, alisma, and Cassia seed not only elevates the content of monacolin K but also enhances the concentration of other active components, thereby improving the efficacy and reducing the toxicity of the medicinal compounds (Yang et al., 2016). The use of different substrates significantly influences the metabolic profile and efficacy of MFRPs, highlighting their multifunctionality and potential in utilizing various agricultural byproducts (Wu et al., 2023). This approach aligns with sustainable production practices, reduces waste, and adds diversity and value to products.

Despite the variety of *Monascus*‐fermented rice products available in the market, it is important to make the choice of dietary supplements according to your physical condition. Although monacolin K is the main blood lipid‐lowering function of MFRP components, the regulation of the body's balance, especially the balance of blood lipids, is by no means a metabolite role, must be its many metabolites of the combined effect, so you can not pursue its content is too high. According to our findings, many of these small molecule metabolites have significant differences in different samples also reflecting the holistic theory of Chinese herbal medicine. For MFRP with high content of monacolin K, such as MR2‐S, MR3‐S are raw materials for the preparation of pharmaceuticals or nutraceuticals, while direct dietary supplements are usually made from MFRP with low content of monacolin K. It was also found that the irradiation form of sterilization was more favorable than the autoclave form of sterilization for the retention and solubilization of small metabolites of MFRP, especially lipids and lipid‐like molecules and amino acids. At the same time, after replacing the substrate of MFRP with highland barley, the metabolite changes are very rich. If you pursue the regulation of blood lipids while considering the need for more nutritional value, the MH product is more mineral elements, amino acids, protein‐based metabolic components, while the MR product is more inclined to lipid‐based metabolic components, which can be selected according to different needs. Although this study provides a reference for the choice of dietary supplements, but also has certain shortcomings. This research and analysis mainly focused on one company's product, meanwhile the substrate of MFRP is now a variety of species, and here only explored barley. The comprehensively research will be carried out in future to continue to move forward.

The results of our current study on MFRP reveal several promising avenues for future research that could significantly enhance our understanding of their potential health benefits and applications in dietary supplements. First, considering the varied biological activities observed among the metabolites identified in different MFRP samples, there is a compelling need to systematically explore these compounds' specific biological functions. Detailed biochemical and molecular biology studies should be conducted to elucidate the mechanisms through which these metabolites exert lipid‐regulating effects and their other potential therapeutic properties. Furthermore, to substantiate the health benefits suggested by our in vitro findings, controlled human clinical trials are essential. These studies should aim to evaluate the efficacy and safety of MFRPs in modulating lipid levels in humans, thereby providing a solid foundation for their use as nutraceuticals or functional foods. Another critical area of research is the impact of different substrates used in the fermentation process of MFRPs. Our study utilized barley; however, exploring a wider range of substrates could uncover how these variables influence the metabolic profiles and efficacy of MFRPs. Such comparative analyses would contribute to optimizing the production processes and enhancing the therapeutic potential of these products.

## 5 CONCLUSIONS

Our investigation into *Monascus*‐fermented rice products (MFRPs) has yielded profound insights into the intricate interplay of factors, namely, monacolin K content, sterilization methods, and substrate selection. The extensive metabolite profiling uncovered a diverse array of MFRPs, emphasizing the pivotal role of monacolin K synthesis. This study delved into the intricate biochemical pathways governing monacolin K production, with notable implications for fatty acid synthesis. Comparing high‐temperature steam with irradiation sterilization methods revealed significant differences in preserving key metabolites, favoring the latter for its superior retention of essential compounds. This suggests a potential paradigm shift toward irradiation sterilization in MFRP processing. The substitution of traditional rice substrate with highland barley induced a substantial shift in the metabolite spectrum of MFRPs, enhancing nutritional profiles and offering new possibilities for tailoring properties to specific health and therapeutic benefits. This study significantly advances the understanding of factors influencing the metabolic profile of MFRPs, paving the way for optimized production techniques and formulation strategies in the dynamic realms of food and pharmaceutical sectors.

## AUTHOR CONTRIBUTIONS

**Yongxia Zhao:** Data curation (equal); formal analysis (equal); software (equal); writing – original draft (equal). **Mingxia Luo:** Software (equal); writing – original draft (equal). **Qin Jiang:** Validation (equal). **Yuhan Ma:** Validation (equal). **Xiaoqi Liu:** Formal analysis (equal). **Xue Bai:** Data curation (equal); formal analysis (equal); methodology (equal). **Lihong Zhou:** Methodology (equal); validation (equal); visualization (equal). **Jian Xie:** Supervision (equal); validation (equal); visualization (equal); writing – review and editing (equal).

## FUNDING INFORMATION

The study was funded by Science and Technology Fund of Guizhou Provincial Health Commission (gzwkj2023‐513), Science and Technology Program of Zunyi (ZSKHHZ [2022]380), and Innovative projects of college students in Guizhou Province (ZYDC2020022).

## CONFLICT OF INTEREST STATEMENT

The authors have declared no conflict of interest.

## ETHICS STATEMENT

This article does not contain any studies with human or animal subjects.

## Supporting information


Table S1.

Table S2.

Table S3.

Table S4.

## Data Availability

The authors confirm that the data supporting the findings and conclusions of this study are available in the article and its supplementary material.
